# Spiculated Bladder Calculi: The Culprit for Repeated Catheter Failure

**DOI:** 10.1155/2013/346891

**Published:** 2013-07-30

**Authors:** C. Wek, T. P. Fox, G. H. Muir

**Affiliations:** King's College Hospital, London SE5 9RS, UK

## Abstract

We report on the case of a frustrated 90-year-old gentleman who was seen in the Accident and Emergency department for the third time in four days with failure of his long-term urethral catheter. He reported that the catheter simply “fell out” with the balloon deflated. On each occasion previously, the catheter had been reinserted in A&E and the patient discharged home. These repeated visits to A&E were understandably a source of much frustration for the patient and his family. On the third presentation, plain abdominal radiography demonstrated a large spiculated bladder calculus.

## 1. Background

Bladder stones account for approximately 5% of urinary calculi and are usually associated with benign prostate enlargement, spinal cord injury, or long-term indwelling catheters [1]. We report on an unusual complication of a bladder stone which caused repeated catheter failure and recurrent urinary retention.

## 2. Case Presentation

A 90-year-old gentleman attended Accident and Emergency department three times in one week with acute retention; each time his long-term catheter had “fallen out.” He had sustained a type 3 odontoid peg fracture five months prior to his attendance, managed conservatively but requiring a long-term indwelling catheter. He was initially referred to A&E by his rehabilitation centre after his indwelling catheter fell out and he went into retention. He was discharged from the A&E after a catheter change. The following day he returned to the A&E with retention and catheter dislodgement, again being discharged after a catheter change. 

Two days later, the patient was brought back to A&E by an ambulance with acute abdominal pain. His catheter had dislodged at the rehabilitation centre and a district nurse was called to resite the catheter. After resiting the catheter, the nurse reported that she heard a balloon burst and so did not attempt to site another. A catheter was inserted by the urology registrar which drained 800 mLs of clear urine. This instantly failed. An abdominal X-ray was requested by the urology registrar.

## 3. Investigations

**Figure 1:**
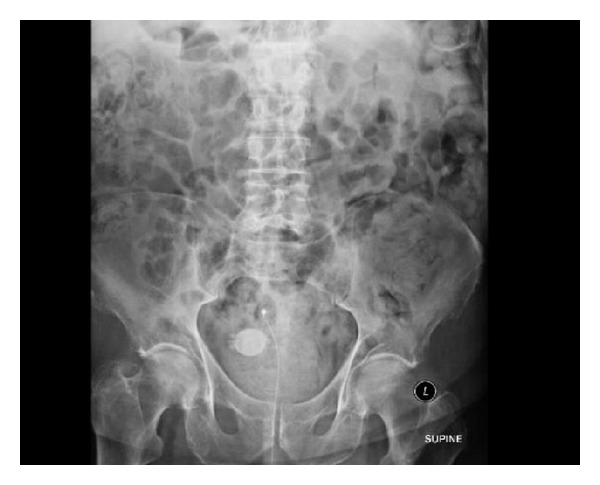
Plain abdominal radiograph clearly showing spiculated bladder calculi.

**Figure 2:**
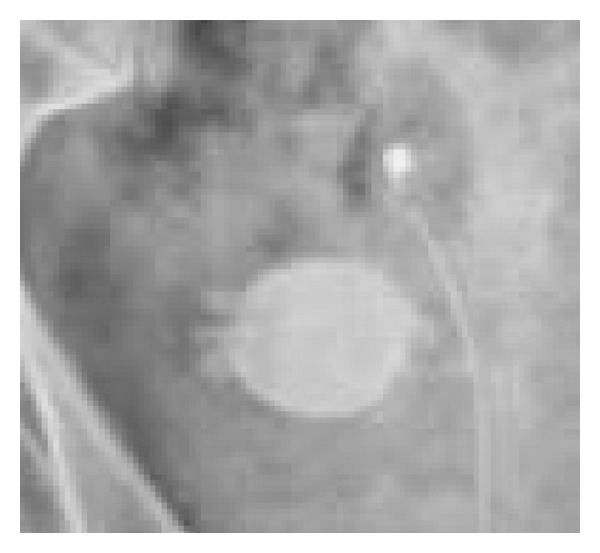
Magnified film showing spiculated bladder calculi.

A plain abdominal X-ray revealed a 3.3 cm spiculated bladder calculus (Figures 1 and 2).

## 4. Differential Diagnosis

The causes of urinary retention can be systematically divided into acute and chronic. Causes of acute urinary retention include urinary tract infections, general anaesthesia and surgery, various medications to treat urinary incontinence such as antimuscarinics, bladder calculi, and neurological causes such as spinal cord compression. There are also causes related to long-term catheter malfunctions such as displacement, blockage, or kinking. 

Causes of chronic urinary retention include benign prostatic hypertrophy, prostate cancer, prostatitis, congenital urethral valves, phimosis, and urethral strictures.

## 5. Treatment

Initially, the patient was recatheterised and the urinary catheter was secured in place with micropore tape instead of relying on an inflated balloon. 

An anaesthetic assessment was carried out with a view to undergoing endoscopic cystolitholapaxy. There were obvious potential hazards in anaesthetising this patient, not in the least his conservatively managed odontoid peg fracture. 

Endoscopic cystolitholapaxy and suprapubic catheter insertion were carried out the following day with no immediate complications.

## 6. Outcome and Follow-Up

Unfortunately, the postoperation period was not completely free of complications. The patient developed a hospital acquired pneumonia which required a course of intravenous antibiotics and an extended stay in hospital. However, he was successfully discharged 10 days later and has had no further issues related to urinary retention to this date.

## 7. Discussion

Urinary retention due to catheter dislodgement is an unusual complication of a bladder calculus. Bladder stones are associated with many complications including recurrent urinary tract infections (most commonly *Proteus mirabilis*), haematuria, and lower urinary tract symptoms (LUTS) [2]. We do not know why adult bladder stones, so common centuries ago, are now very rare [3]. Bladder stones are often more like a hedgehog rather than a smooth pebble in appearance, so it is not hard to imagine how they would burst a catheter balloon.

Most catheter-related problems are managed either in the community by trained district nurses or by emergency department staff. 

Modern Foley catheters are very reliable, and if there is balloon failure more than once, then a bladder stone should be suspected. Suprapubic catheters have a lower rate of symptomatic UTI than urethral catheters and are generally to be preferred if there is no option other than long-term catheterisation for patients.

Bladder stones are certainly not a new phenomenon. Evidence for their existence can be traced back as far as 4800 B.C. to Egyptian mummies. The notable 17th century diarist Samuel Pepys was himself plagued by a bladder stone and described his ordeal in his exquisite detail. Stones afflicted Pepys from an early age and continued to trouble him to the extent that he eventually decided to undergo a lithotomy in 1658. In a time before the germ theory and of course in the absence of antibiotics, this procedure was indeed extremely of high risk. Pepys survived and cited the skill of an early urologist, the prayers of his family, and his own strong constitution as key factors [4].

## 8. Conclusion

This case has identified bladder stones as a possible cause of urinary catheter failure and highlighted the importance of considering imaging in such circumstances. This case also high-lights that suprapubic catheters may well offer a better long-term solution than urethral catheters.
